# Feasibility, potency, and safety of growing human mesenchymal stem cells in space for clinical application

**DOI:** 10.1038/s41526-020-0106-z

**Published:** 2020-06-01

**Authors:** Peng Huang, Athena L. Russell, Rebecca Lefavor, Nisha C. Durand, Elle James, Larry Harvey, Cuiping Zhang, Stefanie Countryman, Louis Stodieck, Abba C. Zubair

**Affiliations:** 1 Department of Laboratory Medicine and Pathology, Mayo Clinic, Jacksonville, FL USA; 2 Center for Regenerative Medicine, Mayo Clinic, Jacksonville, FL USA; 3 Center for Applied Space Technologies, Merritt Island, FL USA; 4 BioServe Space Technologies, University of Colorado Boulder, Boulder, CO USA

**Keywords:** Stem-cell research, Preclinical research, Translational research

## Abstract

Growing stem cells on Earth is very challenging and limited to a few population doublings. The standard two-dimensional (2D) culture environment is an unnatural condition for cell growth. Therefore, culturing stem cells aboard the International Space Station (ISS) under a microgravity environment may provide a more natural three-dimensional environment for stem cell expansion and organ development. In this study, human-derived mesenchymal stem cells (MSCs) grown in space were evaluated to determine their potential use for future clinical applications on Earth and during long-term spaceflight. MSCs were flown in Plate Habitats for transportation to the ISS. The MSCs were imaged every 24–48 h and harvested at 7 and 14 days. Conditioned media samples were frozen at −80 °C and cells were either cryopreserved in 5% dimethyl sulfoxide, RNAprotect, or paraformaldehyde. After return to Earth, MSCs were characterized to establish their identity and cell cycle status. In addition, cell proliferation, differentiation, cytokines, and growth factors’ secretion were assessed. To evaluate the risk of malignant transformation, the space-grown MSCs were subjected to chromosomal, DNA damage, and tumorigenicity assays. We found that microgravity had significant impact on the MSC capacity to secrete cytokines and growth factors. They appeared to be more potent in terms of immunosuppressive capacity compared to their identical ground control. Chromosomal, DNA damage, and tumorigenicity assays showed no evidence of malignant transformation. Therefore, it is feasible and potentially safe to grow MSCs aboard the ISS for potential future clinical applications.

## Introduction

According to the US Census Bureau’s Projections, by the year 2040, 25% of the US population will be 65 years and older^1^. Therefore, the incidence of age-related diseases such as dementia, neurodegenerative diseases, stroke, and cancer will continue to increase. Modern medicine is evolving to address the challenges of this rapidly increasing public health burden. Currently, the most effective method to treat diseased and/or failed organs is organ replacement through transplantation. However, there are not enough donor organs available to meet the needs of the growing number of patients on the waiting list. Expansion of stem cells for regenerative medicine therapies remains a challenge^2^. With the extraordinary growth of regenerative medicine, the demand for stem cells is greatly increasing. Stem cells in general are quiescent and maintain tight control of their numbers within tissues. It is generally believed that stem cells constitute <1% of cells in any given organ^3^.

When induced to proliferate in culture, stem cells can activate pathways involved in differentiation or senescence and may lose their stemness phenotypes after prolonged ex vivo propagation^3^. Most growth media can barely expand stem cells, and if they succeed, it tends to be a slow process. Expansion of the large numbers of stem cells needed for clinical applications is challenging, and standard culture medium formulations are limited in their ability to recapitulate physiological growth conditions^4^. Since the force of gravity is pervasive and affects virtually all aspects of human physiology, we assessed the feasibility, potency, and safety of expanding stem cells under true microgravity conditions for human application.

In microgravity, time can appear to pass faster and as a result humans in space age slower because of the time-dilation effect^5^. Also, exposure to cosmic radiation is much higher in space than on Earth. On average astronauts on the International Space Station (ISS) received about 10 times more radiation than people on Earth^6^. Therefore, aging and radiation-induced organ damage is a health hazard, especially in long duration spaceflight, and is a major concern when humans attempt to colonize other planets. Since organ donors will be very scarce in space, the need for regenerative therapies in this setting will become paramount.

Mesenchymal stem cells (MSCs) are multipotent cells known to modulate immune cell activation and induce tissue repair and promote regeneration^7^. MSCs are fibroblast-like adherent cells that primarily differentiate into osteocytes, chondrocytes, and adipocytes^8–10^. They secrete a broad range of cytokines and growth factors that promote regeneration of other tissue-resident stem cells, such as skeletal, hematopoietic, and neural stem cells. MSCs are identified in vitro by their plastic adherence and expression of surface markers, such as STRO-1, CD73, CD90, CD105, CD271, and CD146, as well as lack of expression of mature hematopoietic lineage markers such as those expressed by B, T, and myeloid cells. They have been used safely in preclinical and clinical studies to modulate inflammation and induce tissue repair, although the mechanisms are not completely understood^11–14^. We recently showed that interleukin-6 (IL-6) and vascular endothelial growth factor play critical roles in MSC-induced neuro-regeneration and anti-inflammation^15^. The involvement of IL-6 is perplexing since IL-6 has been well characterized as a pro-inflammatory cytokine.

MSCs are known to respond to applied- or cell-generated mechanical forces^16^. MSC’s role in mechano-transduction mechanisms associated with bone repair and regeneration has been extensively studied on Earth and to a lesser extent in real and simulated microgravity^17–20^. Unfortunately, it has been challenging to compare the data generated from these experiments because of variation in MSC culture methods, the extent of population doubling or passaging, use of different growth factors, tissue source, and donor-to-donor variation. Also, many studies utilized MSC cell lines, while others used primary cultures. As such, the data generated using each of these highly variable approaches may not be comparable.

There are conflicting reports regarding MSC proliferation characteristics under simulated microgravity^21–23^. This can be attributed to varying experimental approaches, which include different types of microgravity simulators, the use of micro-carriers, levels of oxygenation, and culture media. There are many types of microgravity simulators; the most common is a rotating wall vessel. The rotating wall vessel may not provide true microgravity conditions, rather it works by altering the direction of gravity with respect to the sample over time, or generating rotational centrifugal forces counteracting the gravitational force^18,19^. Therefore, spaceflight experiments like our own are greatly needed to further understand the impacts of real microgravity on MSCs.

Despite the limited value of the data generated from simulated microgravity experiments mainly using animal MSCs, it can be inferred from these studies that microgravity inhibits the growth of MSCs by arresting the cells in the G0/G1 phase of the cell cycle and diminishes the cellular response to growth factor stimulation. Also, microgravity limits MSC’s capacity to undergo osteogenic differentiation through regulation of RUNX2 activity and enhances adipogenic differentiation by upregulating PPARγ2 activity^24^.

The immediate need for stem cells for regenerative therapies is on Earth. The Food and Drug Administration released stringent guidelines regarding the use of cellular products for therapeutic purposes outlined under Code of Federal Regulations 1271. These standards are universal regardless of the source and method used to expand the cells. All cell-based products intended for human use must have established critical quality attributes to ensure identity, purity, sterility, and potency of the product. Tumorigenic potential of the culture-expanded cells must also be evaluated. This is paramount considering the radiation exposure risk in space is several times higher than the risk on Earth.

In this study, we evaluated the feasibility and safety of growing MSCs for human application at the ISS. We have established the identity, purity, viability, and sterility of the space-grown cells compared to ground controls. We have further assessed the functional characteristics of the space-grown cells and assessed their tumorigenic potential. Overall, we have established the feasibility and safety of MSCs grown on the ISS for human application.

## Results

### MSCs maintain their phenotype and proliferative characteristics after expansion on ISS

**Fig. 1:**
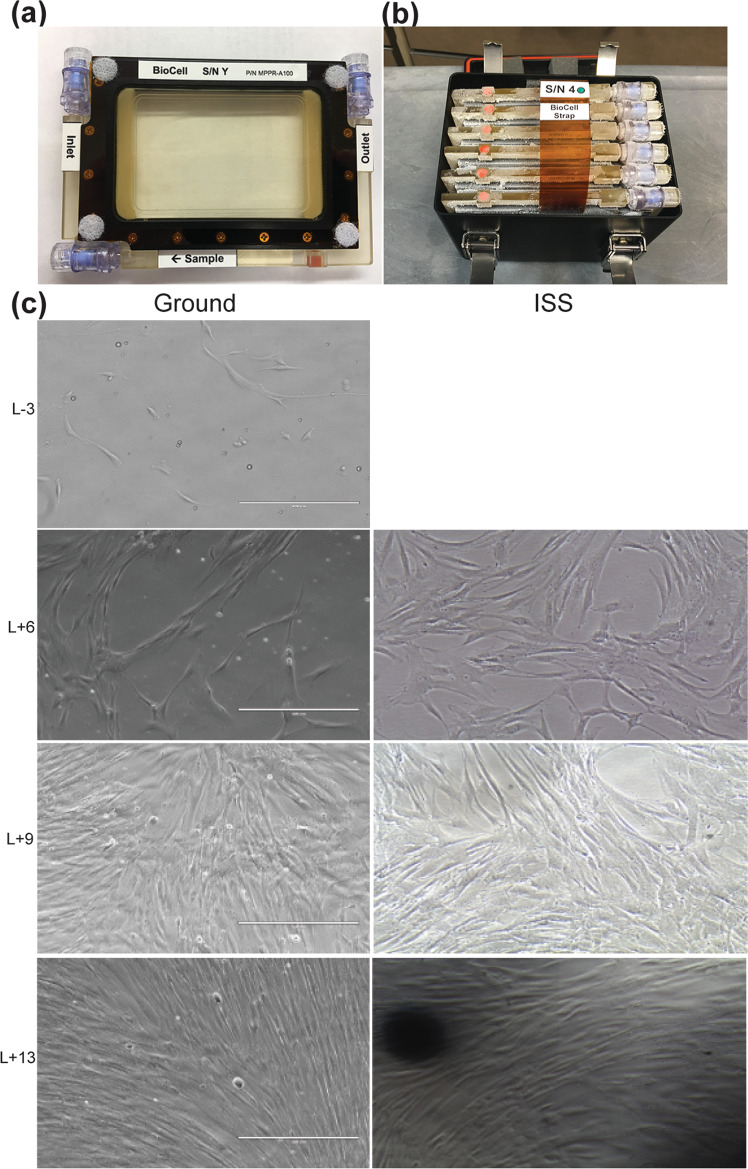
Cell culture and real-time imaging. MSCs were seeded and cultured in BioCells cassettes (**a**) and transported in Plate Habitats (PHABS) (**b**). Real-time images were taken from ground control and ISS microgravity groups. Imaging started 3 days before launching (L − 3) until 13 days after launching (L + 13) (**c**). Scale bar: 400 µm.

MSCs were seeded in BioCell cassettes (1500 cells/cm^2^) 3 days before launch and kept in Plate Habitats (PHABs) at 37 °C. Cell imaging was initiated at ISS 2 days after launch. Cells were imaged in real time at multiple time points for 2 weeks and images were beamed down to Earth (Fig. 1). The ground control cells were imaged simultaneously in our lab. Real-time images of MSCs at ISS showed no obvious differences in morphology and proliferation rate when compared with ground controls. MSCs were cryopreserved in 5% dimethyl sulfoxide and kept frozen at <−95 °C on the ISS until they were returned to Earth using a SpaceX return capsule that landed in the Pacific Ocean. Cells were shipped to our laboratory at Mayo Clinic Florida within 24 h by FedEx.

**Fig. 2:**
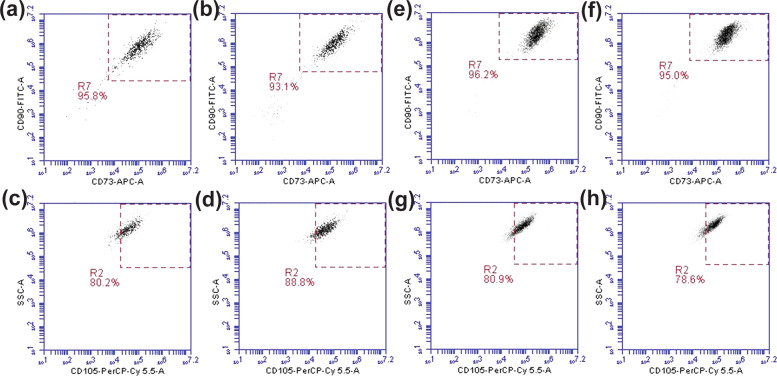
Establishing MSC identity. MSCs thawed from frozen BioCells after 1 week (**a**–**d**) and 2 weeks in culture at ISS were tripled stained with CD73, CD90, and CD105 antibodies and then analyzed using flow cytometry. **a**, **c**, **e**, **g** were from ground control samples. **b**, **d**, **f**, **h** were from ISS microgravity samples. R7 gate was used to select double positive for CD90 and CD73. R2 gate was applied out of R7 gate to demonstrate triple-positive cells for CD73, CD90, and CD105 (*n* = 3).

In our lab the space-expanded MSC (sMSCs) were carefully thawed and we reaffirmed their MSC identity, purity, sterility and post-flight proliferation rate compared with the ground control MSCs (gMSCs). Upon thawing, the mean viability of sMSC was comparable to that of gMSCs (Table 1). This low viability of cells could be attributed to the freezing technique, which did not involve the use of controlled rate freezing. To assess sterility, gram stain and cultures were performed and found to be negative for both sMSCs and gMSCs. Following the immunophenotypic criteria set forth by the International Society for Cellular Therapy^25^, both sMSCs and gMSCs were stained and analyzed for expression of CD73, CD90, and CD105, and lack of CD34, CD11b, CD19, HLA-DR, and CD45 expression by flow cytometry. Our analysis showed that both sMSCs and gMSCs had comparable expression of triple-positive markers (CD73+, CD90+, CD105+) and absence of mature hematopoietic lineage markers (Fig. 2 and Table 1). Fluorescence-activated cell sorting sequential gating strategy has been provided as Supplementary Fig. 1.

**Table 1:** Summary of MSC growth characteristics.

	Pre-flight	Post flight	Post flight	Post flight	Post flight
Duration in culture (week)	1	1	1	2	2
Gravity	Ground	Ground	ISS	Ground	ISS
Identity % (CD73+, CD90+, CD105+)	89.19	80.2	80.9	88.9	78.6
Viability %, mean ± SD	96 ± 1	60.5 ± 14.9	53.1 ± 12.7	67.4 ± 5.1	54.3 ± 20.2
Mean cell count × 10^5^ per BioCell	0.2–0.5	4.25 ± 2.01	5.12 ± 1.21	5.73 ± 1.80	5.11 ± 2.24
Sterility (Gram stain and culture)	Negative	Negative	Negative	Negative	Negative

**Fig. 3:**
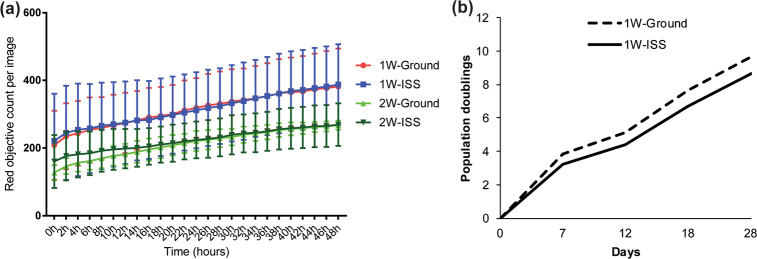
Post-flight MSC proliferation analyses. **a** MSCs were cultured in triplicates after 1- or 2-week expansion in the ISS environment and on Earth in our laboratory. Cells were seeded into a 96-well plate and stained with IncuCyte Nuclight red to track cell proliferation by nucleus count. Real-time images were acquired by IncuCyte S3 at 2-h interval up to 48 h. Post flight, the ISS-expanded cells proliferated at comparable rate with ground control cells. Statistics determined by one-way ANOVA (*n* = 3). Error bars represent standard deviation (SD) (*n* = 3, *P* > 0.05). **b** Similarly, assessment of population doubling in longer-term cultures of 1-week ISS-expanded MSCs and the corresponding ground control showed no statistically significant difference in growth rate (two-sample Wilcoxon’s rank-sum (Mann–Whitney) test, *P* = 0.68). Error bars represent standard deviation (SD).

To assess MSC proliferation potential after space travel, sMSCs and gMSCs were further cultured to quantify their relative growth rates. We determined that both sMSCs and gMSCs have comparable proliferation rates (Fig. 3).

### Spaceflight environment induces changes to cell cycle checkpoint gene expression

**Fig. 4:**
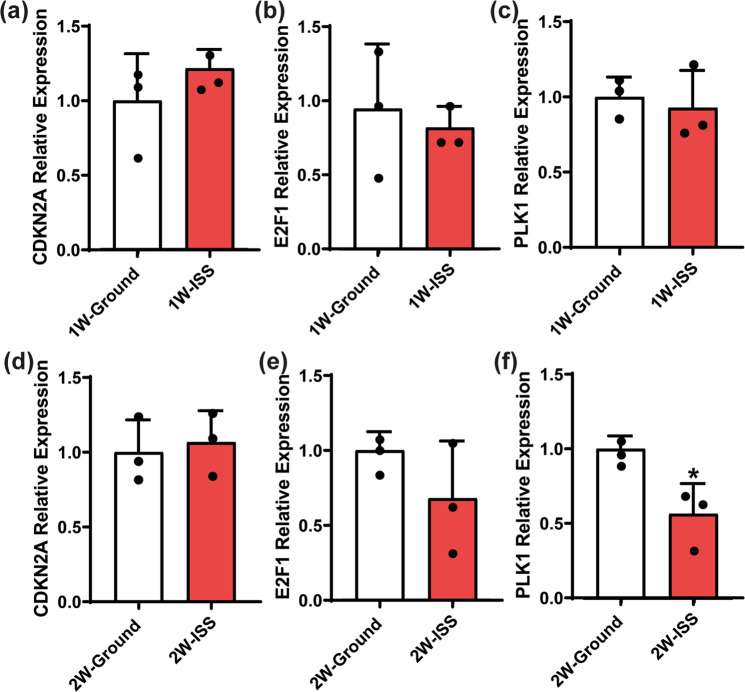
Impact of microgravity on expression of cell cycle checkpoints associated genes. MSCs were culture for 1 and 2 weeks. Cells were harvested and saved in RNAprotect. RNA was later isolated and quantitative reverse transcription PCR (RT-qPCR) analysis was performed as described in the “Methods” section. Expression of cell cycle checkpoint-associated genes: **a** cyclin-dependent kinase inhibitor 2A (*CDKN2A*), **b** transcription factor (*E2F1)*, and **c** serine/threonine-protein kinase, known as polo-like kinase 1 (*PLK1*) were evaluated and compared to ground control gMSCs. Gene expression was normalized to *GAPDH*. Statistics determined by one-way ANOVA (*n* = 3, *P < 0.05), error bars represent standard deviation (SD).

Cells preserved in RNAprotect were subjected to RNA isolation and real-time PCR for cell cycle checkpoint gene expression analysis (Fig. 4). Compared to gMSCs, the expression of *CDKN2A*, a G1/S checkpoint inhibitor, was not significantly different in sMSCs after 1 or 2 weeks in culture. This suggests that within 7–14 days culture, microgravity had minimal impact on MSC capacity to enter into the S phase of cell cycle by altering the expression of *CDKN2A*. E2F1 protein is another cell cycle checkpoint inhibitor that preferentially binds to retinoblastoma protein pRB in a cell cycle-dependent manner, thereby promoting entry into S phase. Our analysis showed that *E2F1* expression by sMSCs may be decreased in microgravity environment, although this decrease was not significant. Polo-like kinase 1 (PLK1) is a serine/threonine-protein kinase that facilitates the transition from G2 to M phase of the cell cycle. PLK1 promotes maturation of the centrosome and establishment of the bipolar spindle. Our analysis showed that *PLK1* expression by MSCs in culture was decreased in microgravity environment, but this decrease was only significant after 2 weeks in culture on the ISS. In summary, it appears in a 7-day culture, microgravity does not significantly alter the expression of *CDKN2A* or *E2F1* or *PLK1*. However, with a 14-day culture, microgravity appeared to downregulate the expression of *PLK1*. Therefore, microgravity conditions may slow the progression of MSCs at later stages of the cell cycle during longer-term culture, but this does not appear to reduce their overall growth rate relative to ground controls. Further studies will be needed to fully validate this finding.

### Prior microgravity exposure does not affect MSC differentiation capacity

**Fig. 5:**
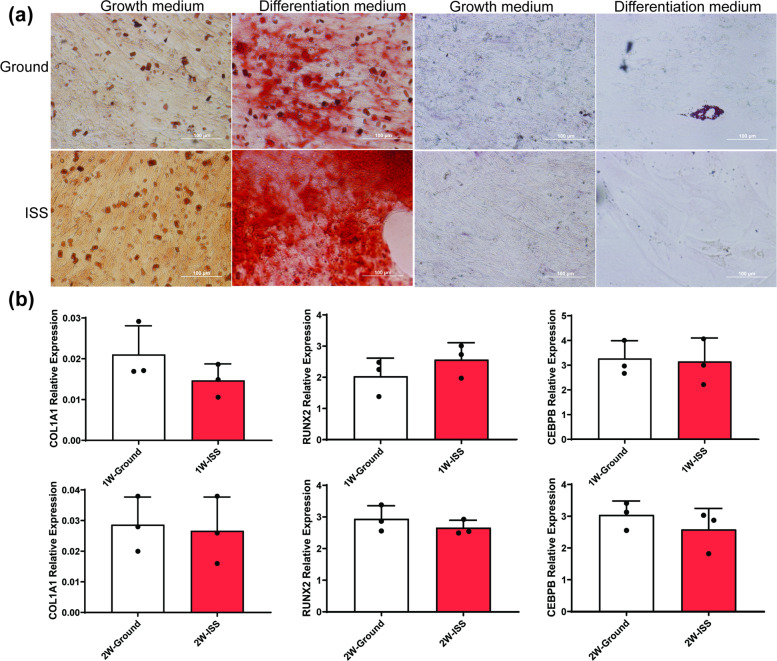
Influence of microgravity on osteogenic and adipogenic differentiation. sMSC and gMSC cultures were stained with Alizarin Red and Oil Red O (**a**). Each condition was seeded and measure in triplicates. Representative pictures (×100) are shown from post-flight MSC culture in a growth medium or differentiation medium after 2-week culture expansion at ISS or ground (Earth). Quantitative real-time PCR analysis of osteogenic (*COL1A1*, *RunX2*) and adipogenic (*CEBPB*) genes was done after 21 days in differentiation medium (**b**). RNA from three 1-week and 2-week MSC cultures were used for quantitative PCR analysis. Gene expression was normalized to *GAPDH*. Statistics determined by one-way ANOVA (*n* = 3). Error bars represent standard deviation (SD). Scale bar: 100 µm.

Osteogenic and adipogenic differentiation assays performed in our lab after the space-grown cells were returned to Earth demonstrated that sMSCs and gMSCs retained similar capacities to differentiate into bone and adipose tissues, as shown with Alizarin Red S and Oil Red O stains, respectively (Fig. 5a). To further investigate the effect of microgravity exposure on MSC differentiation, we analyzed the expression of genes associated with osteogenic (*COL1A1* and *RUNX2*) and adipogenic (*CEBPB*) differentiation by quantitative reverse transcription PCR. Our analysis showed that the expression of the selected genes in space-grown MSCs was statistically similar to ground controls (Fig. 5b). This suggests that MSC expansion for up to 2 weeks in microgravity environment does not affect their capacity to differentiate into osteoblasts and adipocytes.

### Spaceflight environment altered MSC capacity to secrete cytokines and growth factors

**Fig. 6:**
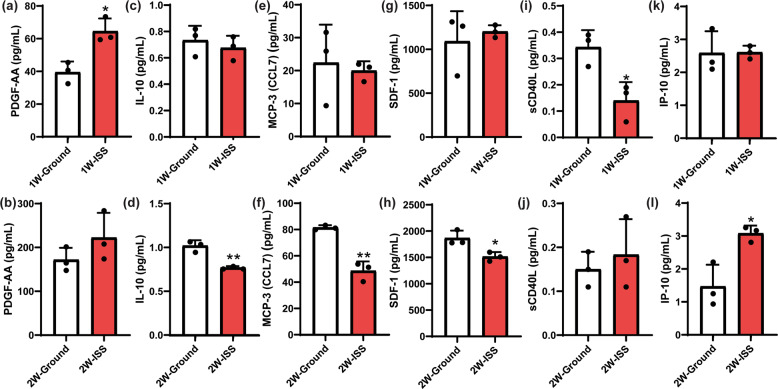
Conditioned media from sMSC and gMSC cultures were evaluated for cytokine and growth factor secretion profile. PDGF-AA (**a**) was increased and sCD40L (**i**) was decreased significantly after 1 week in microgravity. IL-10 (**d**), MCP-3 (**f**), and SDF-1α/β (**h**) were decreased and IP-10 (**l**) was increased significantly in microgravity environment after 2 weeks in microgravity environment relative to ground controls. Statistics determined by Student’s *t* test (*n* = 3, *P < 0.05 **P < 0.01). Error bars represent standard deviation (SD).

Conditioned media from sMSC and gMSC cultures were evaluated for cytokine and growth factor secretion profiles. Sixty-five cytokines and growth factors were evaluated. Secretion of six cytokines and growth factors were identified to be significantly altered by microgravity environment (Fig. 6). Platelet-derived growth factor-AA subunit (PDGF-AA), a potent growth factor known to induce regeneration and tissue repair, was significantly increased and sCD40L, a powerful immunosuppressant, was decreased significantly after 1 week in microgravity. IL-10, a potent T-helper type 2 (Th2) cytokine with tolerance-inducing properties, macrophage chemotactic protein-3 (MCP-3), and stromal cell-derived factor-1α/β (SDF-1α/β), a powerful chemokine known to recruit stem cells and immune cells to sites of injury, were significantly decreased, while IFN-γ-inducible protein (IP-10) was significantly increased after 2 weeks in microgravity environment relative to ground controls. These observations suggest that the effect of microgravity on the secretion of certain cytokines and growth factors by MSCs is dependent on the duration of exposure to microgravity conditions.

### Microgravity environment enhanced the immunosuppressive capacity of MSCs

**Fig. 7:**
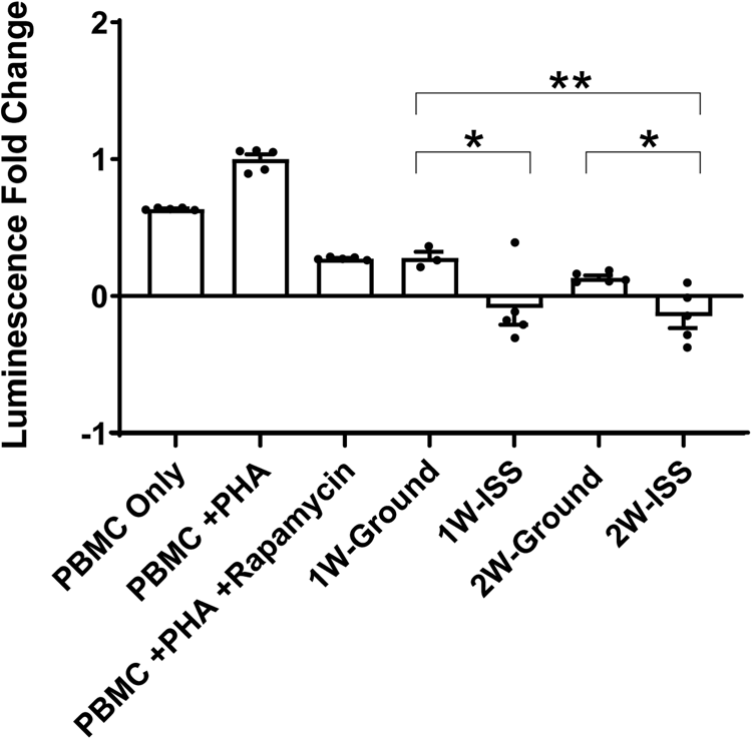
Immunomodulation potency assay comparing the immunosuppressant capacity of gMSC and sMSC towards peripheral blood mononuclear cell (PBMC). MSCs were incubated for 72 h with phytohemagglutinin (PHA) stimulated PBMCs at 1:3 ratio of MSCs to PBMCs. Luminescence is proportional to the amount of ATP present, and the amount of ATP is proportional to the number of metabolically active cells present. Luminescence fold change is relative to the positive control (PBMCs with PHA). Each condition was seeded and measured in quintuplicate. Statistics determined by one-way ANOVA (*n* = 5, *P < 0.05, **P < 0.005). Error bars represent standard deviation (SD).

To evaluate the immunosuppressive capacity of sMSCs compared to gMSCs towards peripheral blood mononuclear cells (PBMCs) in vitro, MSCs were incubated with phytohemagglutinin (PHA)-stimulated PBMCs (Fig. 7). The relative luminescence units (RLUs) from MSCs and PHA-P control wells were subtracted from the RLU measurements from experimental wells to correct for ATP produced by MSC metabolism. RLU measurements from the wells containing PBMCs and PHA-P served as a positive control, representative of the maximum PBMC proliferation. This T cell proliferation assay was assessed by the amount of ATP content, which was proportional to the number of metabolically active cells. Rapamycin served as a positive control and could be observed to suppress the PHA-stimulated T cell proliferation. Compared to gMSCs, sMSCs cultured for 1 or 2 weeks in microgravity were significantly more immunosuppressive as evident by less luminescence signal and therefore less amount of ATP in the co-cultured wells. This observation is not due to differential proliferation rates of MSCs as two immunomodulation potency assays using MSCs from the same donor, with one assay involving irradiated non-proliferating MSCs and the other not irradiated (proliferating), yield the same result (see Supplementary Fig. 2).

### Growing MSCs at the ISS for up to 2 weeks does not compromise genomic integrity

To further assess the safety of growing MSCs in space under microgravity conditions, we determined the presence and extent of DNA damage. This determination was made by visualization and subsequent quantification of the phosphorylation of histone variant γH2A.X at serine 139, in the nuclear compartment of cells. Phosphorylation of H2A.X at S139 is one of the initial signaling events occurring in cells in response to DNA double-strand breaks (DSBs)^26^, and is therefore a well-established marker for DNA damage^27^.

**Fig. 8:**
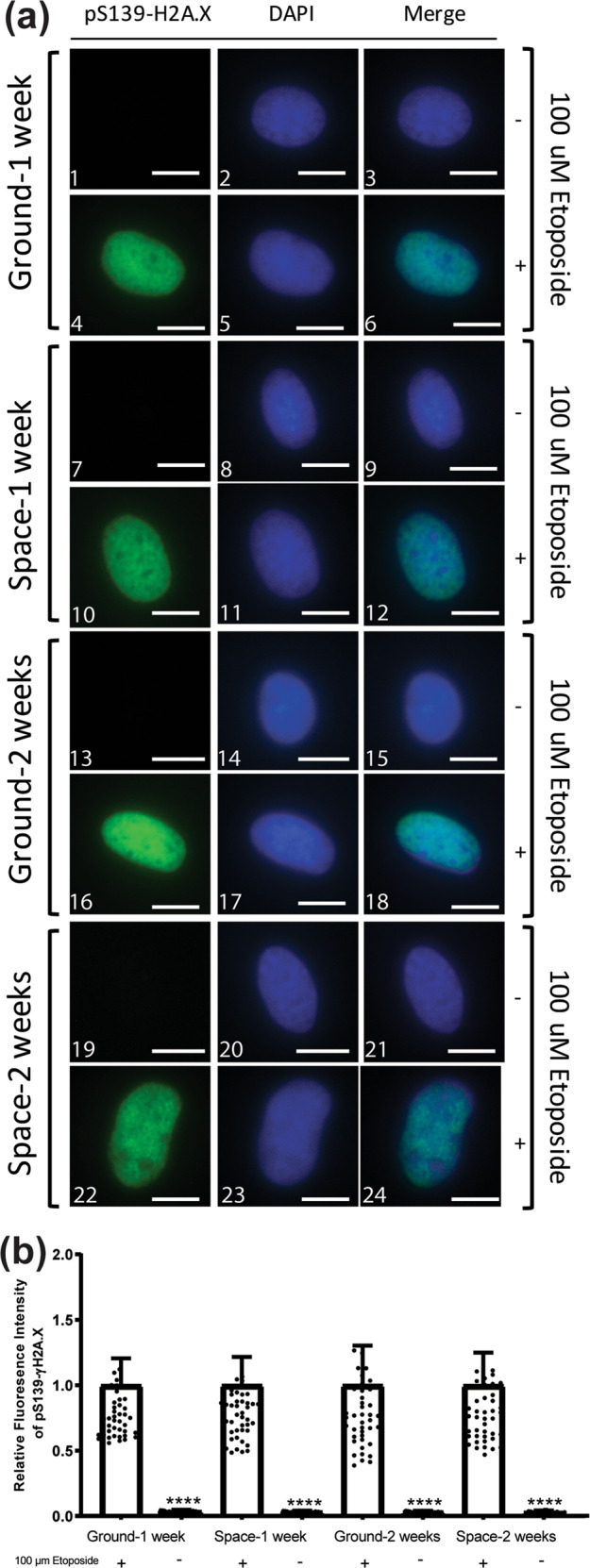
Genomic integrity analysis of the safety of growing MSC in space. **a** Bone marrow-derived mesenchymal stem cells (5000 cells/well) previously grown for 1 week or 2 weeks in space, along with ground control cells were seeded to ibidi µ-Slide 8 Well. Cells were stimulated with 100 μM etoposide as indicated, fixed, and subjected to immunofluorescence analysis to visualize pS139-H2A.X. Scale bars indicate 10 μm. **b** shows quantitation of the fluorescence intensity of pS139-H2A.X (*n* = 40 cells). Analysis was conducted using the Image J software. Fluorescence intensities were normalized to the etoposide-treated group for each experimental condition. **** indicates statistical significance (<0.0001) determined by one-way ANOVA as compared to cells treated with 100 μM etoposide. Error bars represent standard deviation (SD).

Using immunofluorescence, we could not detect pS.139-γH2A.X in the nuclei of cells from any of the experimental conditions evaluated; “Ground-1 week” (Fig. 8a1), “Space-1 week” (Fig. 8a7), “Ground-2 weeks” (Fig. 8a13), and “Space-2 weeks” (Fig. 8a19). As a positive control, a group of cells from each experimental condition was stimulated with 100 μM etoposide (an inducer of DNA DSB) for 2 h, and the presence of pS.139-γH2A.X in the nucleus of these cells was readily detectable (Fig. 8a4, 10, 16, 22). For each experimental condition, there was a significant difference in the nuclear content of pS.139-γH2A.X between cells treated with etoposide and untreated cells (compare Fig. 8a1 with a4, a7 with a10, a13 with a16, and a19 with a22).

Similarly as shown in Fig. 8a, quantification of pS.139-γH2A.X fluorescence intensity (Fig. 8b) present in the nuclei of cells showed a significant difference between the etoposide-treated and -untreated cells in each experimental condition. For all conditions assayed, the percentage of pS.139-γH2A.X in the untreated cells was <4% when compared to their etoposide-treated counterparts normalized to 100%. Most importantly, quantification analysis showed that there were no significant differences among the four experimental conditions, with very similar percentages of pS.139-γH2A.X reported for each one: “Ground-1 week”—3.7%, “Space-1 week”—3.4%, “Ground-2 weeks”—3.3%, and “Space-2 weeks”—3.1% (Fig. 8b).

These data demonstrate that the MSC nuclear content of pS.139-γH2A.X, and therefore associated DNA DSBs, is not affected by growth in microgravity conditions for up to 2 weeks.

**Fig. 9:**
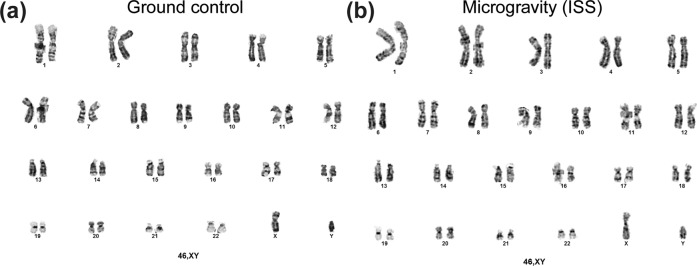
Chromosomal studies were performed using confluent cultures of sMSCs and gMSCs. Twenty cells were analyzed from sMSCs (**a**) and gMSCs (**b**) and two of these were karyotyped using standard G banding method. No chromosomal abnormalities were detected.

In addition, chromosomal studies were performed using confluent cultures of sMSCs and gMSCs. Karyotyping using standard G banding method showed no chromosomal abnormalities (Fig. 9).

### MSCs showed no evidence of tumorigenic transformation after a 14-day culture aboard ISS

**Fig. 10:**
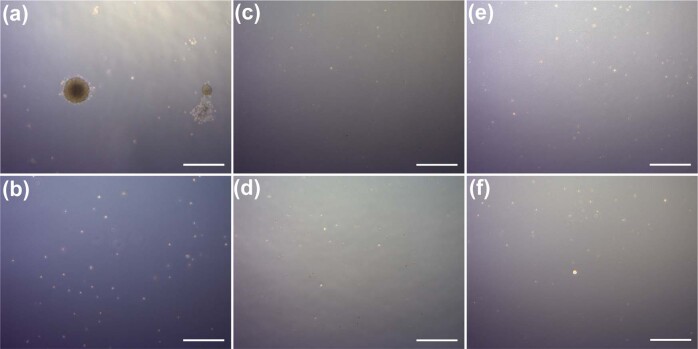
Tumorigenicity assay. Cells were seeded on a soft agar to evaluate their tumorigenic potential. Five thousand cells/well of positive control cell line HT-1080 (**a**) and 67,000 cells/well of negative control cell line W138 (**b**) were used as control to validate the procedure. Twelve cells/well of MSCs were used for this assay. **(c)** Ground control and **(d)** microgravity were cells that were culture expanded for 1 week and (**e**) ground control and **(f**) microgravity were cells cultured for 2 weeks on Earth and in microgravity, respectively.

Although we did not detect any evidence of compromised genomic integrity, there is still concern of tumorigenic transformation due to the presence of cosmic radiation that may result in oncogenic mutation. We therefore performed a tumorigenicity assay to evaluate both sMSC and gMSC tumorigenic potential at 1- and 2-week cultures. HT-1080 was used as a positive control cell line (Fig. 10a) and W138 as negative control cell line (Fig. 10b). Neither sMSCs nor gMSCs showed evidence of tumor formation after a 1- or 2-week culture.

## Discussion

The focus of this study was to evaluate the feasibility and safety of using space-grown MSCs for potential future clinical application on Earth and during long-term spaceflight. Many studies have shown detrimental effects of the absence of gravity on bone homeostasis and the musculoskeletal system. In this study, we aimed to identify the beneficial effects of microgravity on stem cell proliferative capacity with the long-term objective of growing stem cells under microgravity conditions for human regenerative medicine applications. We chose MSCs because they are the most common type of stem/stromal cell used in clinical trials involving cell therapy. A quick query on clinical trials.gov revealed over 10,000 registered clinical trials involving MSC therapy. Our study is unique because it involves the use of primary cultures of human MSCs rather than cell lines. Most of the previous reports using MSCs in microgravity conditions were based on human MSC cell lines or cultured animal-derived MSCs. We therefore believe our study findings reflect as close to reality in space as is practically feasible to studying humans under microgravity.

The first step towards establishing the clinical utility and safety of space-grown cells is ensuring the phenotypic identity of the cells was not changed during growth at ISS under microgravity conditions. Our evaluation showed that MSCs maintained their physical identity and proliferative characteristics aboard ISS. In addition, these physical attributes were maintained after they were returned to Earth and further expanded under standard gravity conditions in our lab. We could not find previous studies that evaluated MSC surface marker identity in real microgravity. However, there were many studies that assessed MSC morphological characteristics under simulated microgravity. Luna et al.^28^ reported that MSCs responded to clinorotation by adopting a more rounded, less-spread morphology.

It was challenging to accurately evaluate MSC proliferation rate in real time while in culture at ISS. Resources at ISS and crew time were the main limiting factors. We resorted to a series of simple microscopic images. These images were sent to us in near real time. In addition, based on a prior experience with fibroblast cell lines, our MSCs had to be seeded on Earth to enable the cells to be adherent, as gravity is apparently needed for this process. Seeding the MSCs at ISS under microgravity may have led to non-adherence and subsequent cell death. We opted to pre-seed the MSCs on Earth. Post-flight MSC count and viability were impacted by cryopreservation method. Overall, our assessment was that the cell counts between ISS-expanded and ground control cells were comparable. Therefore, it is unclear if there is any difference in proliferation rate between the ISS and ground control cells. However, analysis of cell cycle checkpoint gene expression showed that the spaceflight environment after a 14-day culture caused downregulation of *PLK1*, a late G1/S and G2/M checkpoint promoter. This suggests that microgravity could slow MSC proliferation in longer-term cultures. Further studies will be needed to fully elucidate this observation.

Our studies showed that prior microgravity exposure does not affect subsequent MSC differentiation on Earth. However, previous studies of MSC grown in microgravity demonstrated a skewing of their differentiation capacity towards adipogenic lineage^24,29^. Our results do not support these reported studies. Possible explanation as to why our study differs from these reports could be that the effects of microgravity on MSC differentiation to adipogenic lineage require longer microgravity exposure beyond 2 weeks culture. Further studies will be needed to determine if prolonged exposure to microgravity beyond 2 weeks in culture has lasting effects on the MSC differentiation capacity.

Our studies showed that the spaceflight environment altered MSC capacity to secrete certain cytokines and growth factors and this effect was more enhanced with time. MSC capacity to secrete PDGF-AA and IP-10 was significantly increased, while MCP-3 (CCL7), IL-10, sCD40L, and SDF-1 (CCL12) was significantly decreased in microgravity environment relative to ground controls. Contrary to our findings, it was previously reported that microgravity reduces the expression of PDGF-β receptor in rat osteoblasts^30^. Another study reported that human endothelial cells in response to lipopolysaccharide stimulation under microgravity increased the expression of PDGF^31^. MCP-3 and SDF-1 are both potent chemotactic factors known to play significant roles in immune regulation, bone formation, and recruitment of T lymphocytes and monocytes to local inflammatory responses^32–35^. Both immunity and bone formation are known to be altered in microgravity^36–38^. It has been suggested that MCP expression is affected by simulated microgravity. Our study addresses the impact of true microgravity on secretion of MCP-3. Using rat bone marrow-derived MSC, Mitsuhara et al.^39^ reported that CXCR4, a receptor for SDF-1, is overexpressed in simulated microgravity. Similarly, Dr. Kearns-Jonker’s group reported that simulated microgravity induces cardiac progenitor cells (CPCs) to increase SDF-1a messenger RNA (mRNA) expression and this effect was more pronounced in adult CPCs^40^. They also used BioCell Culture Cassette to grow CPCs at ISS and observed similar SDF-1a mRNA overexpression^41^. Although these findings were based on mRNA transcripts in CPC, while ours measured protein secretion by bone marrow-derived MSCs, our study appears to contradict the observation by Dr. Kearns-Jonker’s group. This differential finding highlights the fact that the effect of microgravity varies significantly among different cell types as well as cells at different stages of their life cycles. Also, mRNA expression may not necessarily correlate with protein expression and secretion. More studies will be needed to further investigate these observations.

Our studies showed that microgravity environment enhanced the immunosuppressive capacity of MSCs. It is established that microgravity environment and space radiation significantly affect the immune system^15^. Immune dysregulation in microgravity conditions is not yet fully understood. It is known that cytotoxic T cell function is diminished and this is correlated with reactivation of latent herpesviruses in some crewmembers^42^. Mehta et al.^42^ showed a direct correlation of plasma cytokine alterations with viral shedding in specific crewmembers, and hypothesized that spaceflight is associated with Th2-type tolerogenic immune responses^42^. On the contrary, both sCD40L, a powerful immunosuppressant, and IL-10, a Th2-type cytokine, secretions by sMSCs were significantly decreased in our study. In agreement with these reports, our T cell proliferation assay showed sMSCs to have enhanced immunosuppressive properties via a mechanism that is unlikely to be dependent on IL-10 and sCD40L. Our study highlights a potential role of MSCs in the mechanism of spaceflight-induced immune dysregulation and tolerance. However, further studies will be needed in order to fully understand the mechanism of microgravity-induced immunosuppression.

On the ISS, astronauts receive more than ten times the amount of space and cosmic radiation compared to what humans receive on Earth. This is despite the fact that the ISS orbit does not travel through the Van Allen radiation belts. In contrast, long duration spaceflight outside the Van Allen radiation belts could be very hazardous to humans. This could result in significant DNA damage and genomic instability that may lead to cancer. This is particularly concerning if stem cells are affected. Our studies showed growing MSCs at ISS for 2 weeks does not compromise genomic integrity or evidence of tumorigenic transformation.

Even though we have successfully demonstrated that MSCs can be safely expanded at ISS, our study faced significant challenges and many limitations. Access to ISS is very limited. During the planning and execution of our study, only SpaceX in the United States could provide the required up mass to ISS as well as the desired down mass following the conduct of our research. Other opportunities to access ISS and return payloads to Earth are currently in development and might soon become available. In addition, opportunities for suborbital studies have since become available, such as Blue Origin and the Northrup Grumman Cygnus capsule now regularly launches to the ISS. We were very limited by the weight and volume of our payload. We had to modify our plan to eliminate media exchanges and minimize the amounts of reagents and supplies to be used due to reduced crew time and cold/warm stowage space on ascent to and descent from the ISS. Use of only one donor in our studies is not ideal as we have previously reported that MSC characteristics vary significantly from donor to donor^17^. Crew time is significantly limited as they are tasked with multiple projects. Automated bioreactors that are now offered by multiple implementation partners are very useful and can mitigate some of the challenges highlighted, such as crew time expenditure and complexity of operating equipment at ISS. However, they are very expensive and may consume a significant part of the research budget. We believe the cost will come down once the commercial crew vehicles come online. This will significantly increase available crew time on board the ISS to complete scientific studies such as ours. Our spaceflight studies answered many of our original questions; however, we are now left with more questions than answers and the desire to return to ISS to perform more experiments.

In summary, it is feasible and safe to grow MSCs aboard the ISS. Space-grown MSCs from our study maintained their morphological and phenotypic characteristics, proliferative capabilities, differentiation potential, and were even more potent in their immunosuppressive abilities compared to ground control MSCs. These findings are positive indications that MSCs grown in microgravity could be used for future clinical applications. Our studies demonstrate the feasibility and safety of expanding MSCs at ISS for possible clinical application, but more studies are needed to fully validate our findings.

## Methods

### Experimental design

The experiments utilized BioServe’s single-well BioCell, a cell culture spaceflight certified piece of hardware that can effectively support cell culture. The experiment utilized 17 BioCells containing MSCs were flown in PHABs for transportation to ISS. Once on board the ISS, the PHABs were placed inside of BioServe’s SABL unit, which provides 37 °C temperature control and 5% CO_2_. The experiment also utilized BioServe’s standard inverted phase contrast microscope for imaging of the cells once on board ISS. It also required samples be frozen at <−95 °C once processed and remain frozen until return to Earth. MSC cultures were processed at two time points: at 7 and 14 days. One BioCell was flown specifically for imaging, it was imaged every 48 h for 14 days. MSCs were imaged at ×40 and ×100 magnifications using the VUE camera system from within the microgravity science glovebox. The microscope utilized at the ISS was a standard phase contrast inverted microscope (Nikon TS100). A high-resolution camera attached to the microscope was able to capture both still and video images once the cultures were removed from the on-orbit incubator and placed on the microscope stage. Live pictures were taken for each imaging BioCell periodically and sent to Earth in real time. At the end of the experiment, 5 ml 20% paraformaldehyde (PFA; Electron Microscopy Sciences) (final concentration is 4%) was added to the imaging BioCells directly to preserve the cells at <−95 °C until return to Earth. Similarly, gMSCs were treated exactly the same as those sMSCs in microgravity environment. Even the timing of cell evaluation and cell preservation was approximately maintained. Results of experiments shown in Figs. 3 and 5 involved post-flight Earth re-expanded cells.

### Cell culture and proliferation

Pre-flight mononuclear cells were isolated from a healthy bone marrow donor using Histopaque-1077 (Sigma-Aldrich) following density gradient protocols and used to culture expand the MSCs. The MSCs were cultured in α-minimum essential medium (Fisher Scientific) supplemented with 16.5% fetal bovine serum (FBS; R&D Systems) (designated as Complete Culture Medium, CCM) according to a previously reported method^20,21^. MSCs were expanded to passage 2 and MSC cultures were over 89% pure based on the CD73, CD90, and CD105 co-expression and absence of hematopoietic lineage-specific markers. Expanded MSCs in 25 ml of CCM were seeded into BioCells at passage 4. BioCells were divided into 1- or 2-week groups. The 1-week group was seeded with 5 × 10^4^ MSCs, while the 2-week group was seeded with 2 × 10^4^ MSCs. Identical BioCells were prepared for ground controls. After launch, no media exchange was performed. At the specified time points, MSCs were treated and harvested according to the following procedures. A 4.5 ml media sample was pulled into a monovette through a BioCell sample port and frozen at −95 °C directly for cytokine and growth factor analysis. Cells in the BioCell were trypsinized and 1.5 ml of cell sample was mixed with 5 ml of RNAprotect (Qiagen), and then moved to −95 °C for RNA expression analysis. The remaining cells were frozen in cell preserving medium containing 5% dimethyl sulfoxide (Sigma-Aldrich) directly inside the BioCells and stored at −95 °C.

*Viability post thawing*: Immediately after the BioCells were thawed, aliquots were made for further experiments. Cell number and viability per BioCell were evaluated via Trypan blue (0.4%; VWR) and documented.

*Post-flight real-time proliferation analysis*: Six sMSC and gMSC aliquots from 12 BioCells were selected for real-time cell proliferation analysis post flight. Each aliquot was expanded and 2 × 10^3^ cells/well seeded into a 96-well plate. IncuCyte NucLight Red florescence dye (Essen BioScience) was then added to each well at 1:500 dilution. The 96-well plate-containing cells was loaded into the IncuCyte (Essen Bioscience) with scheduled scanning at 2-h intervals up to 48 h. Automated cells counts from each well were further analyzed using the Prism 8 software (GraphPad).

### MSC phenotyping by flow cytometry

Expanded MSCs were harvested with 0.05% Trypsin (Fisher Scientific). Cells were pelleted at 300 × *g* for 5 min, re-suspended in 2 ml of phosphate-buffered saline (PBS) and filtered through a 35-µm nylon-mesh filter (Fisher Scientific), pelleted again at 300 × *g* for 5 min, and re-suspended to a final volume of 1 ml of PBS. Filtered cells were dispensed to 100 μl aliquots, incubated with MSC marker antibodies against CD73, CD90, and CD105 (BD Biosciences, Human MSC Analysis Kit, Catalog number 562245, Lot number 5313719, 1:100 diluted in BD Pharmingen™ stain buffer), plus antibodies against negative markers CD34, CD11b, CD19, HLA-DR, and CD45^43^. Cells were incubated with antibodies in the dark for 20 min at room temperature. Stained cells were resuspended in a volume of 600 μl with the addition of 500 μl of PBS prior to analysis by flow cytometry.

### Cell cycle analysis

RNA was extracted using RNeasy Plus Mini Kit (Qiagen) as was previously reported^44^ and was prepared for real-time PCR assays. Three biological samples were selected for each group. TaqMan Gene Expression Assays were purchased from Applied Biosystems: GAPDH (Hs02758991_g1), CDKN2A (Hs00923894_m1), E2F1 (Hs00153451_m1), and PLK1 (Hs00153444_m1). TaqMan Fast Advanced Master Mix (Applied Biosystem) were used according to the manufacturer’s user guide. Each sample was tested in triplicate. GAPDH was used as the internal control and the 2^−ΔΔCT^ method was used to analyze data. *GAPDH* is a stable housekeeping gene both on Earth and under microgravity condition^45–47^. For statistical analysis, unpaired *T* tests were carried out using GraphPad Prism 8.

### Osteogenic and adipogenic differentiation assays

Bone differentiation media (BDM) contained CCM plus 1 nM dexamethasone (Sigma-Aldrich), 20 mM β-glycerolphosphate (Sigma-Aldrich), and 50 µg/ml l-ascorbic acid 2-phosphate (Sigma-Aldrich) at a final concentration. Fat differentiation media (FDM) contained CCM plus 0.5 µM dexamethasone, 0.5 µM isobutylmethylxanthine (Sigma-Aldrich) and 50 µM indomethacin (Sigma-Aldrich) at final concentration. MSCs were seeded at 1 × 10^5^ cells/well in 6-well plates. Cultures were propagated in CCM until MSC reached 100% confluence. CCM was then replaced with BDM and FDM for induction of bone differentiation and fat differentiation. Cells were washed with PBS and differentiation media replaced every 3 days for 21 days. After 21 days, media were aspirated and cells were washed with PBS. Two milliliters of 10% formalin (Fisher Scientific) was added to each well and cells were incubated for 1 h at room temperature. Formalin was aspirated and bone differentiation wells were washed with deionized (DI) water, while fat differentiation wells were washed with PBS. One percent Alizarin Red S (Sigma-Aldrich) in DI water was used to stain cells for osteogenesis. For adipogenesis staining, first 0.5% Oil Red O (Sigma-Aldrich) in isopropyl alcohol was diluted with PBS to make a 0.3% working solution. Cells were then stained for adipogenesis with 0.3% Oil Red O. After 20 min at room temperature, the solution was aspirated and cells were washed with DI water and PBS until wash fluid became clear. Each condition was seeded and measure in quintuplicates. Stained cells were then imaged using Olympus inverted microscope at ×100 magnification.

### Quantitative real-time PCR

RNA from MSCs that has been induced under osteogenic and adipogenic differentiation was extracted using RNeasy Plus Mini Kit as was previously reported^45^ and then prepared for real-time PCR assays. Quantitative real-time PCR analysis of osteogenic (*COL1A1*, Hs00164004_m1; *RunX2*, Hs00231692_m1) and adipogenic (*CEBPB*, Hs00270923_s1) genes was done after MSCs have been exposed in a differentiation medium for 21 days.

### Cytokines and growth factor analysis

Cell culture media collected directly from BioCells on the ISS were thawed and aliquoted after we received the payload on Earth. Conditioned medium from three BioCells per time point (1 or 2 weeks in culture) were aliquoted into 200 µl samples. Secreted cytokines and growth factors were analyzed using the Human Cytokine/Chemokine 65-Plex Panel (HD65) (Eve Technologies). Each sample was analyzed in duplicate, and averaged to represent the cytokine/chemokine value. Student’s *t* test was used to compare significance between ground and microgravity (ISS) samples. Details cytokines/chemokines list from Eve Technologies has been included in Supplementary Fig. 3.

### Immunosuppression assay

Cryopreserved MSCs were thawed in a 37 °C water bath and re-suspended in CCM. In white 96-well plates (Greiner, Kremsmünster) MSCs were seeded in quintuplicate at 6.6 × 10^3^ cells/well and stored in a humidified 37 °C, 5% CO_2_ incubator for 2 h. Following incubation, fresh, primary PBMCs (Astarte Biologics) were re-suspended in a medium (RPMI 1650 medium, 5% FBS, 1× Glutamax, 0.05% penicillin/streptomycin; Fisher Scientific) and seeded to wells containing MSCs at 2 × 10^4^ cells/well, resulting in a 1:3 ratio of MSCs to PBMCs. PHA-P (Sigma-Aldrich), a mitogen that activates proliferation of T cells, was added to all wells containing MSCs at a concentration of 10 µg/ml. Additional control conditions were included, which contained PBMCs only, PBMCs with PHA-P, and PBMCs with PHA-P and 10 µg/ml rapamycin (InvivoGen), an IL-2 inhibitor to serve as a functional control of immunosuppression. The plates were incubated in a humidified 37 °C, 5% CO_2_ incubator for 72 h. Following 72 h of incubation, the plates were removed and the quantity of ATP in each well was measured via luminescence using CellTiter-Glo^®^ Luminescent Cell Viability Kit (Promega), which implements a thermostable luciferase that produces luminescent signal proportional to the amount of ATP present in the sample. The RLUs from MSC and PHA-P control wells were subtracted from the RLU measurements from experimental wells to correct for ATP produced by MSC metabolism. RLU measurements from the wells containing PBMCs and PHA-P served as a positive control, representative of the maximum PBMC proliferation. The normalized data was presented as a fold change in comparison to the positive control condition (PBMCs with PHA-P). Statistical significance was determined by one-way analysis of variance, comparing the means of each condition.

### Chromosomal analysis

Chromosomal karyotype was prepared from adherent MSC in a T25 flask grown to confluence. At this point, 25 µl of 1 µg/ml colcemid solution (Sigma-Aldrich) per 2 ml of media was added to the flask and cells were incubated at 37 °C with 5% CO_2_, 5% O_2_, and 90% N_2_ for 16–22 h. The medium was removed from the flask and placed in a 15 ml centrifuge tube and TrypLE Express was used to dissociate the cells. Harvested cells were added to the 15 ml centrifuge tube and centrifuged at 300 × *g* for 8 min. The medium was removed and the pellet was re-suspended in 10 ml of 50:50 hypotonic solution (potassium chloride 0.075 M:sodium citrate 0.8%) and incubated at RT for 20–30 min. Two milliliters of 3:1 fixative (methanol:glacial acetic acid) was added and cells were centrifuged. Pelleted cells were re-suspended in 10 ml of 3:1 fixative (methanol:glacial acetic acid). Centrifugation and addition of fixative was repeated two more times. Cells were dropped on pretreated microscope slides at 25 °C and 65% relative humidity. Twenty cells were analyzed and two of these were karyotyped using standard G banding method.

### DNA damage assay

Bone marrow-derived MSCs (5 × 10^3^ cells/well) were seeded to µ-Slide 8 Well (ibidi) grown to ~70% confluency and stimulated with 100 μM etoposide (Abcam) for 2 h as indicated. Following etoposide treatment, cells were washed three times with PBS and fixed with 4% PFA (37 °C, 15 min). After fixation, cells were washed three times with PBS, permeabilized with 0.1% Triton-X 100 in PBS for 4 min (RT), and blocked with 3% bovine serum albumin and 0.05% Tween-20 in PBS (blocking solution) for 1 h at RT. Samples were incubated with pS.139-H2A.X antibody (Cell Signaling Technology) in blocking solution at 1:250 for 24 h at 4 °C, washed five times with PBS and further incubated with goat anti-rabbit immunoglobulin G (H + L) secondary antibody (Fisher Scientific) at 1:800 and 4′,6-diamidino-2-phenylindole (Sigma-Aldrich) at 1:5000 in blocking solution for 2 h at RT. Subsequently, cells were washed five times with PBS, mounting media (ibidi) was added to the wells, and images were captured using the EVOS FL Cell Imaging System (Fisher Scientific).

### Tumorigenicity assay

Soft agar assay was used to evaluate tumorigenicity of cells grown in microgravity as well as ground controls. Human fibrosarcoma cell line HT-1080 (ATCC, CCL-121) and fibroblast cell line WI38 (ATCC, CCL-75) were used as positive and negative controls. Cells were suspended in 2 ml of top agar (0.35% agar in α-minimum essential medium containing 20% FBS) and then plated on 3 ml of bottom agar (0.5% agar in α-minimum essential medium containing 20% FBS) in a 6-well plate. Colonies were counted after a 14-day incubation.

### Reporting summary

Further information on research design is available in the Nature Research Reporting Summary linked to this article.

## Supplementary information

Supplementary Data

Reporting Summary

## Data Availability

The authors declare that the data that support the findings of this study are available within the paper.
