# Supplementary material for: Feasibility, potency, and safety of growing human mesenchymal stem cells in space for clinical application
Source: NPJ Microgravity. 2020 Jun 1;6:16. doi: 10.1038/s41526-020-0106-z (PMC7264338; doi:10.1038/s41526-020-0106-z)
Supplement: Supplementary file 1 — Supplementary Data [file 41526_2020_106_MOESM1_ESM.pdf]

Supplementary Data

Supplementary Figure 1

Flow Cytometry Gating strategy

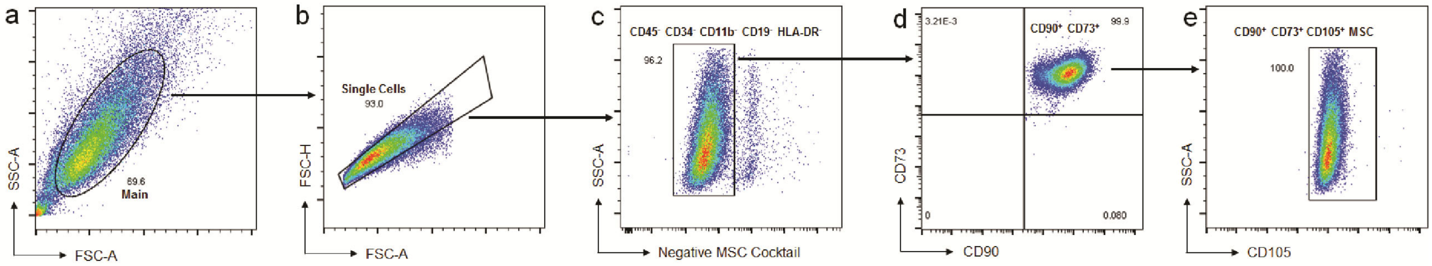

Gating strategies for MSC characterization: First we gate on the main cell population out of total cells acquired (a), and then we exclude doublets by gating on single cells (b). From the single cell population, cells negative for CD45-, CD34-, CD11b-, CD19- and HLA-DR- cocktail of antibodies is selected (c). From the negative staining population we first select double positive for CD73 and CD90 (d). Finally from the CD73/CD90 positive population we gate on CD105 positive to select the triple positive staining (CD73/CD90/CD105) as MSC phenotype markers (e).

Supplementary Figure 2

Human Cytokine/Chemokine 65-Plex Panel (HD65) Eve Technologies):

EGF, Eotaxin, FGF-2, Flt-3 ligand, Fractalkine, G-CSF, GM-CSF, GRO, IFN-α2, IFN-γ, IL-10, IL-12 (p40), IL-12 (p70), IL-13, IL-15, IL-17A, IL-18, IL-1ra, IL-1α, IL-1β, IL-2, IL-3, IL-4, IL-5, IL-6, IL-7, IL-8, IL-9, IP-10, MCP-1, MCP-3, MDC (CCL22), MIP-1α, MIP-1β, PDGF-AA, PDGF-AB/BB, RANTES, TGFα, TNF-α, TNF-β, VEGF, sCD40L, Eotaxin-2, MCP-2, BCA-1, MCP-4, I-309, IL-16, TARC, 6CKine, Eotaxin-3, LIF, TPO, SCF, TSLP, IL-33, IL-20, IL-21, IL-23, TRAIL, CTACK, SDF-1, ENA-78, MIP-1d, IL-28A.

Cytokines/Chemokines from the panel which are not shown in this table were below level of detection.

	G1W-ave	G1W-sd	I1W-ave	I1W-sd	G2W-ave	G2W-sd	I2W-ave	I2W-sd
**FGF-2 (13)**	24.77	1.76	22.01	3.01	64.54	40.35	27.13	1.54
**TGF-a (15)**	0.91	0.43	0.80	0.05	0.66	0.33	0.67	0.15
**G-CSF (18)**	2.57	0.85	2.22	0.95	2.72	1.09	3.52	0.44
**Flt-3L (19)**	3.87	0.88	3.75	0.29	4.26	0.49	4.66	0.12
**GM-CSF (20)**	2.21	0.38	2.54	0.23	4.39	1.26	4.66	1.09
**Fractalkine (21)**	36.95	7.21	38.51	5.79	30.64	4.44	34.85	2.02
**IFNa2 (22)**	6.25	2.56	6.08	1.23	8.05	1.94	6.97	0.91
**IFNy (25)**	2.94	0.09	2.53	0.24	2.99	0.00	3.22	0.37
**IL-10 (27)**	0.73	0.11	0.68	0.09	1.02	0.06	0.77	0.02
**MCP-3 (28)**	22.38	11.56	19.98	2.84	81.57	1.69	48.62	7.11
**IL-12P40 (29)**	5.67	1.53	4.42	0.52	3.53	0.75	4.97	1.42
**MDC (30)**	22.16	4.67	21.90	3.49	17.51	5.29	18.89	5.11
**IL-12P70 (33)**	1.00	0.63	0.42	0.17	0.64	0.27	0.86	0.33
**PDGF-AA (34)**	39.49	6.56	64.58	7.76	171.78	27.42	222.39	56.38
**IL-13 (35)**	0.19	0.12	0.27	0.10	0.21	0.16	0.18	0.08
**IL-15 (37)**	0.84	0.26	0.68	0.18	1.05	0.05	1.20	0.26
**sCD40L (38)**	0.34	0.06	0.14	0.07	0.15	0.04	0.18	0.08
**IL-1RA (42)**	129.43	12.60	129.31	4.84	131.57	3.35	126.12	5.33
**IL-1B (46)**	1.60	0.28	1.54	0.13	1.97	0.20	1.85	0.32
**IL-4 (53)**	2.29	0.99	1.89	0.78	1.66	1.46	2.34	0.89
**SDF-1a+B (64)**	1091.72	342.53	1202.94	71.70	1866.88	142.14	1515.91	85.25
**IL-6 (57)**	1500.94	500.21	1658.05	582.26	3674.27	680.14	4447.71	924.37
**IL-7 (61)**	3.30	0.55	3.58	0.25	4.04	0.17	4.10	0.17
**IL-8 (63)**	94.87	15.26	93.13	12.46	116.83	9.63	121.85	22.57
**IP-10 (65)**	2.59	0.66	2.62	0.28	1.47	0.66	3.08	0.24
**MCP-1 (67)**	9869.75	5108.60	9151.18	1167.20	26600.34	5983.04	22821.63	4523.00
**MIP-1B (73)**	3.98	0.11	3.60	0.65	3.23	0.65	3.56	0.16
**RANTES (74)**	5.36	1.35	5.39	1.12	6.37	0.78	6.53	1.65
**VEGF-A (78)**	391.01	40.37	355.36	20.31	300.31	29.56	262.04	27.57
**IL-18 (66)**	3.85	0.35	3.62	0.38	3.68	0.40	3.73	0.46
**MCP-2 (13)**	4.38	0.31	4.35	0.33	2.91	0.75	3.42	0.29
**LIF (34)**	64.31	29.04	75.52	4.71	121.18	7.12	117.05	16.06
**TPO (36)**	15.23	7.45	18.26	7.22	18.51	6.07	14.98	6.62

Unit for all data: pg/ml
G1W-ave: Average of 3 individual samples from 1 week ground control group
G1W-sd: STDEV of 3 individual samples from 1 week ground control group
I1W-ave: Average of 3 individual samples from 1 week Microgravity group
I1W-sd: STDEV of 3 individual samples from 1 week Microgravity group

G2W-ave: Average of 3 individual samples from 2 weeks ground control group
G2W-sd: STDEV of 3 individual samples from 2 weeks ground control group
I2W-ave: Average of 3 individual samples from 2 weeks Microgravity group
I2W-sd: STDEV of 3 individual samples from 2 weeks Microgravity group

Supplementary Figure 3

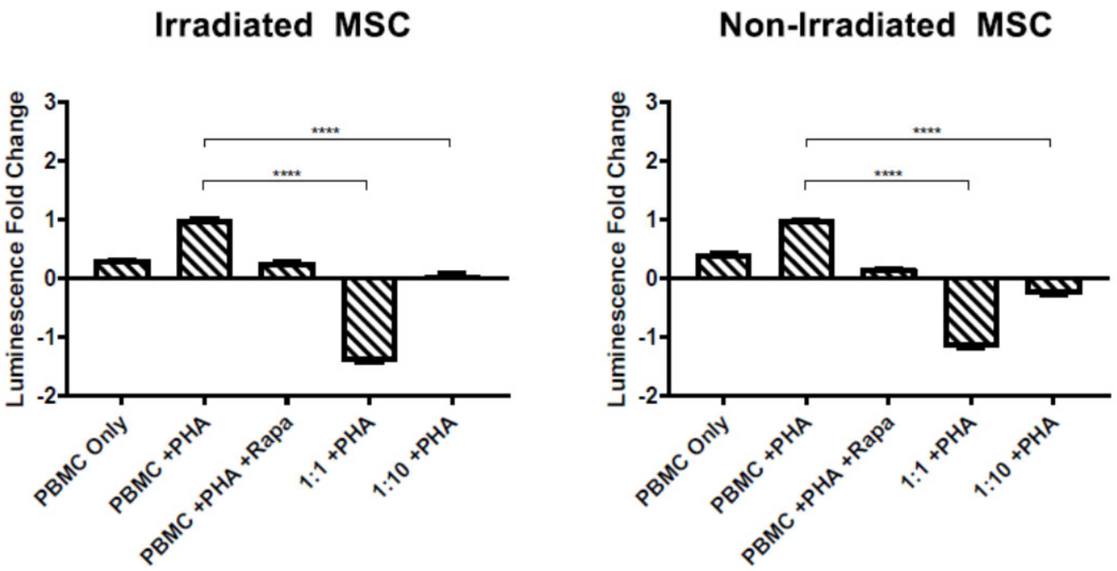

Immunomodulation potency assay comparing the immunosuppressant capacity of mesenchymal stem cells (MSC) towards peripheral blood mononuclear cells (PBMC). Irradiated and non-irradiated MSCs were incubated for 72 hours with phytohemmaglutinin (PHA) stimulated PBMCs at a 1:1 and 1:10 ratio of MSCs to PBMCs. Luminescence is proportional to the amount of ATP present, and the amount of ATP is proportional to the number of metabolically active cells present. Luminescence fold change is relative to the positive control (PBMCs with PHA). Each condition was seeded and measured in quintuplicate. Statistics determined by one-way ANOVA. **** $p<0.0001$
